# AI foundation models for RNA biology

**DOI:** 10.1080/15476286.2026.2650517

**Published:** 2026-03-24

**Authors:** Haopeng Yu, Yiliang Ding

**Affiliations:** Department of Cell and Developmental Biology, John Innes Centre, Norwich Research Park, Norwich, UK

**Keywords:** Foundation model, RNA biology, Pre-training, fine-tuning, explainable AI (XAI), RNA sequence-structure-function

## Abstract

RNA biology is undergoing a transformative revolution driven by AI foundation models. These models learn the intricate relationships between RNA sequence, structure, and function by training on vast, diverse datasets spanning millions of RNA molecules across various species. Through self-supervised learning on these sequences, these models acquire a generalizable understanding of RNA, which can then be fine-tuned for various downstream tasks, thereby enabling the decoding of functional rules embedded in RNA sequences. In this review, we provide a comprehensive guide to RNA foundation models. Using concrete examples of RNA biology, we begin with the concept of foundation models and review the importance of pre-training datasets, architectural innovations, self-supervised strategies, and fine-tuning approaches that allow general RNA representations to be translated into task-specific models. Crucially, we highlight how explainable AI (XAI) methods transform these models from black-box predictors into valuable discovery tools that reveal candidate cis-regulatory elements and structural motifs. As RNA foundation models keep advancing and integrating more multimodal biological data, they aim to uncover additional regulatory rules and functions encoded in RNA.

## 1. Introduction

Over the past several decades, humanity’s capacity to generate and collect data has expanded dramatically. However, this explosion of information has also been accompanied by increasingly intricate internal relationships within these ‘big datasets’. In response, the quest to enable machines to learn patterns from complex, high-dimensional data, and thereby uncover the rules they encode, has driven the evolution of artificial intelligence (AI) [1]. AI has progressed from classical machine learning (ML), through the transformative era of deep learning (DL), and ultimately towards the rise of modern AI foundation models, which surpass previous paradigms by offering a unified and highly generalizable strategy for learning from vast and heterogeneous datasets [2–4]. This breakthrough was first demonstrated in generative AI models, like GPT models, trained on human language, enabling natural, conversational interaction and giving rise to today’s widely adopted AI agents and AI assistants. In the area of biology, researchers have been applying foundation model strategies to decipher the underlying rules of molecular biology. In the protein domain, protein foundation-model-based approaches, such as ESM-2/ESM-3 and AlphaFold 3, have achieved highly accurate structural predictions, outperforming earlier generations of protein modelling tools and fundamentally transforming our ability to model protein structure [5,6].

In RNA biology, foundation-model-based approaches are only beginning to emerge, but they are rapidly reshaping our ability to decode RNA sequence – structure – function relationships. RNAs do far more than carry the coding blueprint for proteins, they also embed rich post-transcriptional ‘regulatory grammar’ that governs how transcripts are translated, how quickly they are degraded, and how they interact with RNA-binding proteins and chemical modifications such as RNA methylation [7–9]. Moreover, RNA is an intrinsically structural molecule [10]. Through Watson–Crick and non-canonical base pairing between reverse-complementary segments, it folds into secondary and higher-order conformations that create binding surfaces, catalytic pockets, and regulatory switches, enabling diverse and sometimes highly complex biological functions [11–13]. These properties make RNA an especially compelling target for foundation models, which can learn joint representations of sequence and structure from large unlabelled datasets and thereby support generalizable predictions across RNA families and functions.

**Figure 1.**
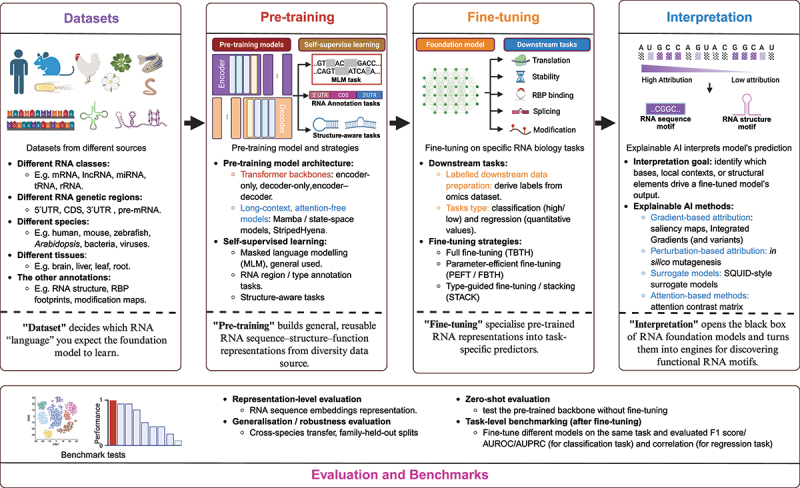
Overview of RNA foundation models in RNA biology. The schematic summarises the RNA foundation model pipeline: diverse RNA datasets drive self-supervised pre-training of general sequence–structure–function representations, which are then specialised by fine-tuning for downstream tasks. It also highlights evaluation benchmarks and explainable-AI strategies that open the black box to pinpoint predictive sequences or structural motifs. Created in BioRender.

In this review, we explore the emerging field of RNA foundation models, discussing their underlying principles, the methodologies used in their pre-training and fine-tuning, and their application to RNA biology (Figure 1, Table 1 and Table S1). We review the datasets that fuel these models, the architectures that drive their performance, and the evaluation strategies that validate their predictions (Figure 1). Additionally, we highlight the role of explainable AI (XAI) in enhancing our understanding of the model’s decision-making process (Figure 1). By synthesizing current advances, this review is tailored for RNA biologists and aims to aid the widespread adoption of foundation models as hypothesis-generation tools. Nevertheless, it is also valuable for machine learning researchers building RNA foundation models to quickly review previous work.

**Table 1.** Overview of RNA foundation models.

Model	Year	Model Params	Datasets	Model Architecture	Self-supervised tasks
Nucleotide Transformer [19]	2025	500 M, 2.5B	Multi-species transcriptome	Encoder-only (Transformer)	Masked Language Modeling (MLM)
UNI-RNA [20]	2024	400 M	RNA	Encoder-only (Transformer)	Masked Language Modeling (MLM)
PlantRNA-FM [32]	2024	35 M	1,124 plant transcriptomes	Encoder-only (Transformer)	Masked Language Modeling (MLM), annotation, RNA structure
GenerRNA [34]	2024	350 M	Mixed RNA sequences	Decoder-only (Transformer)	Causal Language Modeling (CLM)
GARNET [35]	2024	19 M	rRNA	Decoder-only (Transformer)	Causal Language Modeling (CLM)
Orthrus [39]	2024	1–10 M	pre-mRNA	Encoder-only (Mamba)	Contrast Learning
DGRNA [40]	2024	100 M	MARS database (100 M RNA sequences)	Encoder-only (Mamba)	Masked Language Modeling (MLM)
ATOM-1 [37]	2024	Not disclosed	In-house wet-lab assays	Encoder–Decoder (Transformer)	Cross-modal
CodonBERT [26]	2024	87 M	Mammals, bacteria, viruses CDS	Encoder-only (Transformer)	Masked Language Modeling (MLM) and homologous sequences prediction
RNA-FM [21]	2023	100 M	RNAcentral ncRNAs	Encoder-only (Transformer)	Masked Language Modeling (MLM)
AIDO.RNA [23]	2023	1.6 B	Multi-database ncRNAs	Encoder-only (Transformer)	Masked Language Modeling (MLM)
RiNALMo [22]	2023	135–650 M	Aggregated ncRNAs	Encoder-only (Transformer)	Masked Language Modeling (MLM)
UTR-LM [24]	2023	1 M	Endogenous and synthetic 5′UTRs	Encoder-only (Transformer)	Masked Language Modeling (MLM) and auxiliary tasks
CaLM [25]	2023	86 M	Codonized cDNA sequences	Encoder-only (Transformer)	Masked Language Modeling (MLM)
3UTRBERT [27]	2023	86 M	Human 3’UTRs	Encoder-only (Transformer)	Masked Language Modeling (MLM)
RNA-MSM [38]	2023	95 M	RNA MSAs datasets	Encoder-only (MSA-Transformer)	Masked Language Modeling (MLM)
SpliceBERT [28]	2022	20 M	Vertebrate pre-mRNA	Encoder-only (Transformer)	Masked Language Modeling (MLM)

## 2. The basis of foundation models

Foundation models are large-scale models that learn broad, general-purpose representations from massive, diverse datasets and can be efficiently adapted to a wide range of downstream tasks [14]. To illustrate this setting, imagine you are studying mRNA translation efficiency (TE). After deriving TE values from Ribo-seq data, you might ask, ‘What in the RNA sequence makes these TE behave so differently among genes?’ Classic statistical and bioinformatics analyses can reveal many significant correlations. However, these correlations are genome-wide results; they tell you where to look, but not which specific cis-elements or structural motifs within the RNA sequence are driving TE differences. So, you decide to train an RNA foundation model to learn the regulatory ‘code’ of the RNA sequence.

Building an RNA foundation model is typically described in two stages: pre-training and fine-tuning [14,15]. During pre-training, the model is exposed to large collections of diverse unlabelled RNA sequences from many species and is encouraged to ‘read’ and ‘understand’ them, a process known as self-supervised learning [16]. After this stage, the model has acquired a general representation of RNA sequence diversity and context. Fine-tuning then focuses this general knowledge on your specific biological question, ‘the impact of 5′ UTR on translation efficiency’. Here, you provide labelled data, either TE values treated as a continuous variable (a regression task) or binned into high/low TE (a binary classification task). Because the model has already learned rich RNA representations through large-scale pre-training, it can adapt quickly and typically achieve strong predictive performance even with modest amounts of data. Once fine-tuned, the model can be used as an *in silico* experimental partner: you can systematically alter the 5′UTR sequence and observe how the predicted TE changes, an approach often termed *in silico mutagenesis* (ISM) [17,18]. In addition, interpretability methods, known as explainable AI (XAI), can be applied to the fine-tuned model to extract candidate functional RNA sequences or RNA structure motifs that affect translation efficiency [17].

One RNA foundation model can be adapted to many different downstream tasks. If you later wish to examine how the 3′UTR influences TE, you can fine-tune the same RNA foundation model on 3′UTR sequences and repeat the analysis. Likewise, if your interest shifts to RNA stability, RNA-binding protein (RBP) binding, or RNA methylation, the model’s general understanding of RNA provides a strong starting point; you simply fine-tune it using the relevant datasets.

Thus, pre-training equips the model with broad RNA ‘knowledge’, fine-tuning tailors it to a specific RNA biology question, and interpretability reveals the sequence or structural features behind the predictions (Figure 1). This is the essence of the RNA foundation model.

## 3. Dataset for RNA foundation model

Pre-training an RNA foundation model usually starts with large-scale transcriptomic datasets spanning multiple species and tissues (Figure 1). The choice of data at this stage is important: if a study targets a particular RNA class, such as plant RNAs, or only the coding sequence (CDS) regions of mRNAs, then the collected sequences should be constrained accordingly (Figure 1, Table 1). Pre-training on such a focused corpus produces a foundation model specialized for that RNA type or region. For instance, universal RNA models such as Nucleotide Transformer are pre-trained on around 3,202 human genomes and an additional 850 genomes from other species, totalling roughly 174 billion nucleotides [19]. Uni-RNA scales to an even broader corpus sourced from RNAcentral, NCBI and GenomeWarehouse (GWH) databases, totalling about one billion RNA sequences for pre-training [20]. In plant RNA biology, PlantRNA-FM is designed for plant-specific applications and is trained exclusively on plant transcriptomes; its pre-training set spans 1,124 plant species and includes approximately 25.0 million RNA sequences (around 54.2 billion bases). For ncRNA-centred models, pre-training data are likewise restricted to non-coding transcripts. RNA-FM draws from RNAcentral, yielding roughly 23.7 million non-redundant ncRNA sequences across many organism groups [21]. RiNALMo was trained with 36 million non-coding RNA sequences aggregated from several public databases [22]. AIDO.RNA extends this idea further, pre-training on roughly 42 million public ncRNA sequences in a comparable all-lineage mixture [23]. Another RNA region-specific model narrows the corpus to particular mRNA regions. UTR-LM, for example, pre-trains only on 5′ UTRs, combining endogenous 5′ UTRs from multiple species with a synthetic library of random 5′ UTRs [24]. For coding-region specialization, CaLM is trained on codonized protein-cDNA sequences from about 9 million non-redundant genes [25]. CodonBERT also targets CDS, but at codon resolution, and is pre-trained on more than 10 million CDS sequences pooled across mammals, bacteria, and human viruses [26]. Other segment-specific corpora include 3′ UTR models such as 3UTRBERT [27], which pre-trains on the 3′ UTRs of 108,573 human mRNA transcripts, and process-specific pre-mRNA models such as SpliceBERT, which uses around 2 million pre-mRNA sequences from 72 vertebrate species [28]. More recently, multimodal biological foundation models have begun to jointly pre-train on DNA, RNA and protein information, for example, Evo 2 and related cross-modal frameworks, enabling unified representations across the central dogma [29].

## 4. Architectures of the RNA foundation models

Regarding the model architecture, most RNA foundation models are built as Transformer towers, where stacked self-attention layers naturally capture the long-range base – base dependencies common in RNA [30]. In the classical Transformer design, this tower can be organized into an ‘encoder – decoder’ pair, where the ‘encoder’ reads the full input sequence to form contextual representations, and the ‘decoder’ generates or reconstructs sequences step by step from those representations [31] (Figure 1). Encoder-only models process the full input sequence by allowing each nucleotide position to attend to all other positions simultaneously (the bidirectional attention mechanism) and are best suited for prediction tasks such as RNA structure prediction or RBP binding site identification, where rich contextual representations of existing sequences are required. Decoder-only models generate sequences autoregressively and are better suited for RNA design tasks, where the goal is to produce novel sequences with desired functional properties. Many use encoder-only Transformers to produce rich contextual embeddings for each nucleotide (e.g. PlantRNA-FM, ERNIE-RNA and RiNALMo), which is ideal for downstream prediction tasks [22,32,33] (Table 1). A smaller set uses decoder-only Transformers for RNA generation (e.g. GenerRNA and GARNET), where the model learns to ‘write’ RNA sequences [34,35]. Some frameworks explore encoder – decoder Transformers when the goal is to map a sequence to another representation, such as a structural or activity representation (e.g. OPED and ATOM-1) [36,37] (Table 1).

In addition to standard Transformer towers, several models adapt the backbone to better align with biological priors. For example, RNA-MSM is built on an MSA-Transformer that ingests multiple-sequence alignments rather than single sequences, allowing the model to exploit evolutionary conservation and co-variation patterns across homologs directly [38] (Table 1). By contrast, because full self-attention scales quadratically with sequence length, long-RNA settings have motivated long-context sequence backbones beyond quadratic attention. Mamba-based architectures (e.g. Orthrus [39] and DGRNA [40]) replace attention with state-space dynamics that scale linearly and remain effective on very long transcripts, while StripedHyena in LoRNA uses an efficient long-context operator tailored to ultra-long reads [41] (Table 1).

## 5. Pre-training strategy for RNA foundation models

Next, we should design tasks that encourage the model to ‘read’ and ‘understand’ RNA sequences, which are known as self-supervised tasks (Figure 1). Currently, the most widely used self-supervised pre-training task is to mask about 15% nucleotides and let the model fill them back in, purely using the sequence around them, a technique called masked language modelling (MLM) [42]. MLM is preferred when the downstream goal is prediction or biological understanding, as bidirectional context produces richer representations of RNA sequence. RNA foundation models, like RNA-FM, AIDO.RNA, ERNIE-RNA, RNA-MSM, SpliceBERT and RiNALMo all rely on MLM as their core pre-training objective to learn contextual dependencies from raw RNA sequences [21,22,28,33,38,43] (Table 1). Alongside these, several models adopt MLM-based variants to inject extra biological signal. RNABERT combines MLM with an alignment/structure-aware auxiliary task (MLM/SAL) and CodonBERT augments MLM with a homology-related objective at the codon level (MLM/HSP) [26,44] (Table 1). By contrast, causal language modelling (next-token prediction), used in decoder-only models such as GenerRNA and GARNET, is better suited for generative applications such as designing novel RNA sequences with desired functional properties.

Beyond that, pre-training also supports embedding multiple self-supervised tasks. PlantRNA-FM is pre-trained with three self-supervised tasks, including MLM, RNA genetic annotation classification and RNA structure prediction, effectively asking the model to learn RNA sequence, RNA annotation and RNA structure features together [32]. These representations capture which nucleotide patterns tend to occur in RNA, recurring sequence/structure motifs, and long-range dependencies. In short, before we ever expose it to a specific downstream task, the model has already developed a general, biologically grounded understanding of RNA.

Admittedly, pre-training a foundation model is always costly. Beyond the substantial storage and CPU time needed to turn millions of RNA sequences into a ‘pre-training-ready’ dataset, the main bottlenecks lie in the training itself: GPU memory, which limits how large a model you can load, and GPU compute, which determines how quickly you can do the model training. For high-capacity models trained on genuinely large-scale datasets, pre-training can easily run into thousands of GPU-hours, often requiring continuous training over many days or weeks; for example, RiNALMo has been run on 8×A100 for roughly a day at cloud-scale hourly cost and PlantRNA-FM reports pre-training on 4× A100 GPUs for about 3 months, illustrating the sustained compute needed even for RNA foundation model [22,32]. In practice, most researchers do not need to repeat this expense from scratch. Once a powerful, pretrained model is available, it can serve as a starting point and be adapted to new biological questions by fine-tuning on a task-specific dataset. Fine-tuning usually requires only modest computational resources, making it the practical way for most groups to bring foundation-model power into their RNA research.

## 6. Fine-tuning, adapting to downstream RNA tasks

After pre-training, you now have a powerful RNA foundation model with a broad understanding of RNA diversity and context. Next, you can use fine-tuning to guide the RNA foundation model’s focus to the specific RNA biology of interest (Figure 1). At this stage, we need labelled data; in other words, we have to tell the model exactly which function we want it to learn from the RNA sequence (i.e. the downstream task) (Figure 1). Typically, a general-purpose RNA foundation model is evaluated across a broad panel of downstream tasks. For instance, AIDO.RNA was benchmarked on a comprehensive suite spanning 9 task categories, including RNA structure prediction, RNA functional annotation, and mRNA-relevant regulatory tasks, to show that a single pre-trained model can generalize across diverse RNA downstream tasks [23]. PlantRNA-FM was pre-trained solely on plant RNA and designed for plant-specific tasks; therefore, it outperforms other RNA foundation models in plant RNA annotation and plant RNA translation efficiency prediction tasks [32].

Once these RNA downstream tasks are prepared, we can fine-tune the RNA foundation model on the task of interest. The most straightforward fine-tune strategy is called ‘full fine-tuning’, where all model parameters are updated. This often gives strong performance when the fine-tuning data are closely aligned with the pre-training domain. Full fine-tuning is recommended when sufficient labelled data are available, as it typically yields the strongest task-specific performance. The other is parameter-efficient fine-tuning, which keeps most of the pretrained model’s parameters fixed (i.e. freezing layers/encoder/backbone) and trains only a small number of newly added parameters, such as lightweight adapter layers or low-rank updates (LoRA) [45,46]. These strategies make training cheaper and typically more stable, and they reduce the risk of overfitting when the labelled dataset is limited. Where resources permit, we recommend testing both full fine-tuning and parameter-efficient fine-tuning on the same dataset and selecting the approach based on evaluation metrics such as accuracy, F1 score, or Pearson correlation, as appropriate to the task. In RNAErnie, a type-guided fine-tuning framework is proposed, including three variants: FBTH (Frozen Backbone and Trainable Head), TBTH (Trainable Backbone and Trainable Head; equivalent to full fine-tuning), and STACK (type-guided stacking) [47]. In STACK, the backbone is first used to predict a coarse-grained RNA type; the predicted type token is then appended to the end of the sequence, and the model is further fine-tuned on the downstream task [47].

The fine-tuning strategy for an RNA foundation model is similar to that used in deep learning more broadly [3]. Normally, the dataset was split into a training set, a validation set, and a test set (80%, 10%, 10%). Some studies adopt slightly different protocols, for instance, ERNIE-RNA fine-tunes on RNA – protein binding by first holding out 20% of the data as an independent test set, then splitting the remaining 80% into training and validation sets in a 9:1 ratio [33]. In most cases, the objective of fine-tuning encourages the model to make predictions on the unseen validation set that are as close as possible to the ‘ground-truth’ values (i.e. the labels). Strong performance on the validation and test sets suggests that the model generalizes well to new, unseen data, rather than simply memorizing the training set (a problem known as overfitting). Each biological question has its own labelled dataset, is treated as a separate fine-tuning task, and produces a task-specific fine-tuned model.

## 7. Benchmarks and evaluation of RNA foundation models

After pre-training and finetuning, a natural question is: how do we know whether the model has really learned the RNA biology insights? In practice, evaluation is usually carried out at several complementary levels (Figure 1).

At the representation level, a common evaluation method is to input RNA sequences into the pretrained model and extract the embedding layer, producing a high-dimensional matrix. This high-dimensional embedding matrix can be thought of as the model’s ‘understanding’ of RNA. If pre-training has worked well, embeddings from different RNA categories should start to look different: for example, different RNA types (mRNA, tRNA, rRNA), or different regions within the same transcript (CDS and 5′/3′ UTRs). Researchers often visualize these embeddings with dimensionality-reduction methods such as UMAP. For instance, SpliceBERT projects pretrained site embeddings into UMAP space and finds that true branchpoint motifs separate from background sites, suggesting the model has already captured splicing-related sequence patterns [48]. Another widely used strategy is zero-shot testing, in which the pretrained model is asked to tackle a downstream task without any task-specific fine-tuning. RiNALMo, which generalizes to secondary-structure prediction on RNA families unseen during training, both indicating that pre-training alone has encoded useful biological signal [22].

For task-level evaluation, performance is assessed after each RNA foundation model has been fine-tuned on the same downstream task and consistent fine-tuning strategies. Typical task suites for RNA foundation models include RNA structure prediction, RBP binding prediction, splice-site/branchpoint recognition, translation-efficiency classification, RNA-stability (half-life) classification, and RNA modification detection. Reported scores depend on task type. For classification, metrics usually include F1 score, the harmonic mean of precision and recall, as well as AUROC and AUPRC, which summarize performance across decision thresholds. AUROC is widely used, while AUPRC is often more informative when positives are rare, as in many binding or modification datasets. For regression tasks (e.g. quantitative translation efficiency or stability), studies typically report MAE and correlation measures such as Pearson or Spearman. For example, in UTR-LM’s benchmark tests, IRES identification improves AUPRC from 0.37 to 0.52 over the best baseline, and MRL (mean ribosome load) prediction improves Spearman by about 5% compared with MTtrans [24].

Evaluation of generalization is also a crucial component, assessing whether performance remains consistent on unseen datasets. This includes transfer across species, robustness on RNA families not seen during training, and resilience to shifts between experimental platforms or batches. RiNALMo holding out entire RNA families during fine-tuning demonstrates strong inter-family generalization on secondary-structure benchmarks, which is a more stringent test than random splits over sequences [22].

Several benchmark datasets have been developed and used to compare RNA foundation models across tasks. For RNA secondary-structure prediction, widely used resources include bpRNA, ArchiveII and RNAStralign database [49–52]. For RNA 3D structure and structure–task, RNA3DB provides non-redundant, structurally dissimilar splits from PDB chains, and newer multi-dataset benchmarks have begun to compile structure–function tasks in a unified way [53]. BEACON provides a clear community benchmark for RNA foundation models: it curates 13 RNA tasks spanning structural analysis, functional prediction and engineering applications, compares RNA LMs against traditional baselines under a unified fine-tuning/evaluation protocol, and explicitly studies how design choices such as tokeniser granularity and positional encoding affect downstream performance [54]. AIDO.RNA offers a complementary large-scale suite from a model-development perspective, assembling 26 datasets across 9 task categories (covering structure, function, mRNA-related traits such as translation and stability, and design tasks) [43].

## 8. Interpreted RNA foundation model with explainable AI

Explainable AI (XAI) methods are essential if RNA foundation models are to do more than predict labels [17]. In practice, interpretation is about turning a task-specific fine-tuned model into a map of causal candidates: which bases, which local RNA contexts, and which RNA structure elements are driving the output (Figure 1). The most widely used route is attribution, where one estimates the contribution of each nucleotide to the prediction either through gradients (saliency maps, Integrated Gradients and their variants) or through perturbation, often framed as *in silico* mutagenesis (ISM) [55–57]. These two families are conceptually complementary: gradient attribution provides a fast, single-base-resolution view of importance, while perturbation mirrors experimental logic by asking how the prediction changes when a sequence segment is altered or shuffled. Beyond attribution, surrogate modelling frameworks such as SQUID provide mechanistically grounded explanations of cis-regulatory logic [58].

For Transformer-based RNA models, attention and representation analyses provide a third interpretability signal that is particularly natural for problems with long-range dependencies. Attention maps often highlight distant base-to-base relationships or position-specific cues that align with known RNA priors. Even before fine-tuning, ERNIE-RNA’s attention patterns and embeddings show sensitivity to RNA secondary-structure features, suggesting that the backbone already tracks base-pairing and folded context [33]. That kind of built-in structural awareness becomes useful downstream, because one can compare attention-derived contacts or high-attention regions with predicted or experimentally probed structures to justify why a model calls a site functional [33]. PlantRNA-FM provides one of the most explicit demonstrations of this end-to-end logic in RNA foundation models. After fine-tuning for plant translation-related tasks, it uses an attention-contrast strategy, subtracting the attention patterns of a real-label model from those of a random-label control, to identify RNA sequence motif, like KOZAK sequence and 112 functional RNA structure motifs affect translation efficiency [32]. In more universal backbones such as RNA-FM, the interpretability stack tends to follow the “standard FM-XAI’’ pattern: fine-tune to a function (for example, RBP binding or structural tasks), compute gradient or perturbation attribution, and cluster the strongest windows to infer binding or structural motifs [21]. The breadth of RNA-FM’s pretraining means that these inferred motifs can generalize across families and organisms, which is precisely the promise of foundation models once their predictions are made explainable [21].

RNA foundation models learn rich, context-dependent representations during pretraining, but biological insights emerge only when we interrogate those representations with XAI. However, current XAI methods remain imperfect. Each method comes with its own assumptions and trade-offs, and there is no globally optimal strategy for model interpretation [59]. We therefore recommend that researchers apply multiple XAI approaches when interrogating RNA foundation models and validate outputs against established biological knowledge, that is, ‘the ground truth’. Comparing identified motifs with experimentally verified RNA sequence or RNA structure elements (e.g. known RNA sequence motif, known RBP binding sites, and validated RNA structure motif) helps determine which XAI strategy is most effective for a specific biological question.

## 9. Discussion

The development of RNA foundation models has marked a pivotal shift in our ability to understand the complexities of RNA biology. Through the application of self-supervised pre-training on large, diverse RNA datasets, these models learn generalized representations of RNA sequences, structures, and their functional implications. This foundational knowledge allows for the model’s fine-tuning on specific RNA-related tasks, making it highly adaptable and effective across various RNA biology inquiries, from translation efficiency to RNA-protein interactions.

Each step of the foundation model pipeline plays a critical role in its success. Pre-training, often on extensive datasets spanning multiple species, provides the essential backbone for model generalization, allowing the model to learn contextual relationships within RNA sequences that might otherwise remain obscured. Fine-tuning then refines these representations for specific biological questions, enabling the model to predict and explain RNA behaviour in a targeted and highly effective manner. Whether it is predicting translation efficiency based on UTR sequences or uncovering regulatory motifs within RNA-binding protein interactions, fine-tuning leverages the foundation model’s broad RNA knowledge and adapts it to specific experimental contexts.

Evaluation methods, both at the representation and task levels, provide the necessary validation for model predictions. At the representation level, the extraction of high-dimensional embeddings allows researchers to understand the underlying structure of RNA data and how different RNA categories or regions are represented. At the task level, benchmarks across a range of RNA-related tasks, including structure prediction, binding affinity prediction, and stability classification, ensure that the model’s outputs are accurate and reliable. Furthermore, evaluating the model’s generalization capacity – whether across RNA families or experimental platforms – provides confidence in its robustness and ability to tackle a broad spectrum of RNA biology questions.

The true power of RNA foundation models is unlocked through their integration with explainable AI (XAI). By using methods such as gradient-based attribution or *in silico* mutagenesis, researchers can interpret the model’s predictions, identifying key sequence elements or structural motifs that drive observed biological outcomes. These insights bridge the gap between machine learning and traditional molecular biology, offering mechanistic explanations that can guide experimental design and hypothesis generation. This interpretability also enhances the utility of foundation models as experimental partners, providing in silico mutagenesis capabilities to predict the effects of sequence or structural changes on RNA function.

Importantly, advances in AI foundation models should not be seen as a competition between AI and human researchers. Rather, they represent a technological revolution within scientific research. Researchers therefore need to understand the strengths of foundation models and the kinds of problems they are well suited to solving, and to ask how this powerful class of tools might address questions at the heart of their own research interests, especially those that were previously infeasible because of the combinatorial complexity of nucleotide sequences, and because of limitations in experimental scale, cost, or practical complexity. For instance, foundation models can enable the systematic discovery of latent regulatory rules across vast sequence spaces, help to prioritize testable causal hypotheses from noisy data, or allow perturbation landscapes to be explored *in silico* before committing to wet-lab validation. Adopting this mindset reframes foundation models from mere tools into discovery engines, enabling RNA biology to tackle questions and derive answers that were previously out of reach.

In practice, while pre-training RNA foundation models requires expensive, high-performance GPU clusters and substantial computational time, fine-tuning and explainable AI analyses are far more accessible, often achievable on desktop-level GPUs within hours. In most cases, fine-tuning an existing pre-trained RNA foundation model is preferable, particularly when labelled data are limited or when capturing long-range sequence dependencies is important. Training a model from scratch is justified only when the biological question requires incorporating information not present in existing pre-training corpora. For instance, current RNA foundation models are pre-trained primarily on sequence, annotation, and structural information; if a researcher wished to integrate population-level data, such as GWAS data or phylogenetic relationships, into the model’s representations, pre-training a new model that incorporates such data would be necessary. We encourage RNA biologists to fine-tune existing pre-trained models on their own datasets rather than training from scratch, thereby benefiting from generalized RNA representations while minimizing computational costs. We also suggest that foundation model developers provide accessible code, clear documentation, and user-friendly interfaces to lower barriers for adoption.

Looking forward, the future of RNA foundation models is brimming with promise. As datasets grow larger and more diverse, models will continue to improve their ability to generalize across RNA families, species, and experimental conditions. The advent of multimodal models that combine RNA, DNA, and protein information holds the potential to provide even deeper insights into the molecular underpinnings of cellular processes. Moreover, as explainable AI techniques evolve, the interpretability of these models will become even more refined, allowing for more accurate identification of regulatory elements and guiding the development of RNA-targeted therapeutics. Ultimately, RNA foundation models represent a transformative approach to RNA biology, paving the way for more sophisticated and precise studies of RNA function, regulation, and disease.

## Supplementary Material

TableS1.docx

## Data Availability

The authors confirm that the data supporting the findings of this study are available within the article and/or its supplementary materials.
