# Supplementary material for: AI foundation models for RNA biology
Source: RNA Biol. 2026 Mar 24;23(1):1–11. doi: 10.1080/15476286.2026.2650517 (PMC13048551; doi:10.1080/15476286.2026.2650517)
Supplement: TableS1.docx [file KRNB_A_2650517_SM7968.docx]

**Table S1. Download links of RNA foundation models**

| **Model** | **Model Download Link** | **Code Repository** |
| --- | --- | --- |
| Nucleotide Transformer | https://huggingface.co/InstaDeepAI/nucleotide-transformer-2.5b-multi-species | https://github.com/instadeepai/nucleotide-transformer |
| UNI-RNA | - | - |
| PlantRNA-FM | https://huggingface.co/yangheng/PlantRNA-FM | https://github.com/yangheng95/PlantRNA-FM |
| GenerRNA | https://huggingface.co/pfnet/GenerRNA | https://github.com/pfnet-research/GenerRNA |
| GARNET | https://zenodo.org/records/14003346 | https://github.com/Doudna-lab/GARNET_DL |
| Orthrus | https://huggingface.co/quietflamingo/orthrus-large-6-track | https://github.com/bowang-lab/Orthrus |
| DGRNA | - | - |
| ATOM-1 | - | - |
| RNA-FM | https://huggingface.co/cuhkaih/rnafm | https://github.com/ml4bio/RNA-FM |
| AIDO.RNA | https://huggingface.co/genbio-ai/AIDO.RNA-1.6B | https://github.com/genbio-ai/AIDO |
| RiNALMo | https://huggingface.co/multimolecule/rinalmo | https://github.com/lbcb-sci/RiNALMo |
| UTR-LM | https://huggingface.co/multimolecule/utrlm.te_el | https://github.com/a96123155/UTR-LM |
| CaLM | https://huggingface.co/multimolecule/calm | https://github.com/oxpig/CaLM |
| 3UTRBERT | https://huggingface.co/multimolecule/utrbert-3mer | https://github.com/yangyn533/3UTRBERT |
| RNA-MSM | https://zenodo.org/records/8280831 | https://github.com/yikunpku/RNA-MSM |
| SpliceBERT | https://huggingface.co/multimolecule/splicebert | https://github.com/biomed-AI/SpliceBERT |
| CodonBERT | https://github.com/Sanofi-Public/CodonBERT | https://github.com/Sanofi-Public/CodonBERT |
